# Are underprivileged and less empowered women deprived of respectful maternity care: Inequities in childbirth experiences in public health facilities in Pakistan

**DOI:** 10.1371/journal.pone.0249874

**Published:** 2021-04-15

**Authors:** Waqas Hameed, Mudassir Uddin, Bilal Iqbal Avan

**Affiliations:** 1 Department of Community Health Sciences, Aga Khan University, Karachi, Pakistan; 2 Department of Statistics, University of Karachi, Karachi, Pakistan; 3 Department of Clinical Research, London School of Hygiene and Tropical Medicine, London, United Kingdom; 1. IRCCS Neuromed 2. Doctors with Africa CUAMM, ITALY

## Abstract

**Background:**

Attainment of healthcare in respectful and dignified manner is a fundamental right for every woman regardless of the individual status. However, social exclusion, poor psychosocial support, and demeaning care during childbirth at health facilities are common worldwide, particularly in low- and middle-income countries. We concurrently examined how women with varying socio-demographic characteristics are treated during childbirth, the effect of women’s empowerment on mistreatment, and health services factors that contribute to mistreatment in secondary-level public health facilities in Pakistan.

**Methods:**

A cross-sectional survey was conducted during August–November 2016 among 783 women who gave birth in six secondary-care public health facilities across four contiguous districts of southern Sindh. Women were recruited in health facilities and later interviewed at home within 42 days of postpartum using a WHO’s framework-guided 43-item structured questionnaire. Means, standard deviation, and average were used to describe characteristics of the participants. Multivariable linear regression was applied using Stata 15.1.

**Results:**

Women experiencing at least one violation of their right to care by hospital staff during intrapartum care included: ineffective communication (100%); lack of supportive care (99.7%); loss of autonomy (97.5%); failure of meeting professional clinical standards (84.4%); lack of resources (76.3%); verbal abuse (15.2%); physical abuse (14.8%); and discrimination (3.2%). Risk factors of all three dimensions showed significant association with mistreatment: socio-demographic: primigravida and poorer were more mistreated; health services: lesser-education on birth preparedness and postnatal care leads to higher mistreatment; and in terms of women’s empowerment: women who were emotionally and physically abused by family, and those with lack of social support and lesser involvement in joint household decision making with husbands are more likely to be mistreated as compared to their counterparts. The magnitude of relationship between all significant risk factors and mistreatment, in the form of β coefficients, ranged from 0.2 to 5.5 with p-values less than 0.05.

**Conclusion:**

There are glaring inequalities in terms of the way women are treated during childbirth in public health facilities. Measures of socio-demographic, health services, and women’s empowerment showed a significant independent association with mistreatment during childbirth. At the health system level, there is a need for urgent solutions for more inclusive care to ensure that all women are treated with compassion and dignity, complemented by psychosocial support for those who are emotionally disturbed and lack social support.

## Introduction

The World Health Organisation (WHO) states that “every woman has the right to the highest attainable standard of health, which includes the right to dignified, respectful health care” [1]. Yet at health facilities in low- and middle-income countries in particular, women’s experience of maternity care is characterised by discrimination, poor psychosocial support, and service providers. Condescending attitude towards them [2–4]. A women’s experience childbirth can leave life changing psychological scars [5, 6]. Research shows that a third of women describe their experience of giving birth as traumatic [7].and such psychological distress during labour leaves women uniquely susceptible vulnerable to the effects of environmental factors, such as unfamiliar personnel, and medicalised procedures among others [8]. Poor encounters during obstetrical care may adversely affect the quality and outcome of a women’s birthing experiences leading to psychological disorders [9–11] and deterring her from seeking health care in the future [2, 12, 13].

A *Lancet Commission on Global Health* recently highlighted the need for high-quality health systems that improve health, and are valued, trusted, and responsive to dynamic population needs [14]. The *Commission* further emphasised that health services need to be distributed in an equitable manner [14]. the context of maternal health, as in general most work illuminating inequities in health service delivery has focused on access to or the utilisation of services. There is however little evidence around inequities in the quality of maternal care. A mixed-method systematic review identified several socio-economic demographic and health system perpetrators of mistreatment during childbirth in facility-based settings [2]. Several quantitative studies have attempted to examine the inequalities underpinning women’s experiences of this mistreatment these have yielded diverse findings which indicates that risk factors vary according to context.

Women’s empowerment is a multidimensional concept, but is usually linked with their ability to use resources effectively to achieve desired outcomes [15–18]. More specifically, key dimensions of women’s empowerment include: the ability to make decisions and exercise influence, self-perception and personal freedom, access to and control over resources, and support from social networks [19]. Researchers have used various measures alone and in combination to capture women’s (dis)empowerment such as involvement in household decision making (economic, household affairs, fertility desire), intimate partner violence, self-esteem, physical mobility, and social support [20, 21]. While increased empowerment is generally known to have positive effects on women’s health-seeking behaviours and on maternal and child health outcomes [22–24], there is little evidence on how this multifaceted quality may influence a women’s experience at delivery. A landscape review on mistreatment during childbirth identified dearth of quantitative data linking empowerment and experiences of mistreatment [2]. We found two studies from India [25] and Pakistan [26] that showed a protective effect of empowerment on mistreatment. In India, researchers used various dimensions of empowerment as described above; however, their analysis did not take into account potential health system factors that may confound the relationship between empowerment and mistreatment during childbirth. The empowerment measure used in Pakistan’s study was based solely on women’s participation in household decision making while neglecting other dimensions. Mindful of the fact that not all dimensions of women’s empowerment correlate equally with mistreatment, our study assessed various dimensions of empowerment separately in relation to experiences of mistreatment during childbirth.

We noted that in most studies on mistreatment, there were weaknesses in how certain health system and demographic characteristics were measured especially in LMIC settings. For example, the use of average family/household income as opposed to wealth quintile [27]; a dichotomous measure of antenatal care/visit (crude coverage) versus the level of education on regarding different aspects of birth preparedness (effective coverage) [28].

An understanding of inequalities underpinning mistreatment during childbirth is essential for designing appropriate interventions, Yet to the best of the authors’ knowledge the available evidence from South East Asia is inconclusive. Of the two available studies, in Pakistan one suffers from the aforementioned limitations in terms of weak measures [29]; and the other has an element of recall bias in that women who had given birth within the previous 12 months were interviewed [26]. Although, both studies revealed a high prevalence of mistreatment during childbirth, the factors characterising inequalities were inconsistent [26, 29]. In view of the gaps in the available literature, this paper aims to: a) examine whether and how women with varying demographics are treated differently during childbirth; b) capture the role of women’s empowerment in shaping experiences of mistreatment during childbirth; and c) identify health services factors that contribute to mistreatment.

### Country context

With 216 million inhabitants, Pakistan is the fifth most populous country in the world [30]. Nearly two-thirds of the population lives in rural areas [31]. Pakistan has one of the highest rates of maternal mortality [32], and is ranked the riskiest country for newborns [33]. It also has the highest prevalence (range 29–66%) of anxiety and depression among women in Asia [34]. Over the years, the country has recorded a continuous rise in the proportion of antenatal coverage, institutional births, and uptake of postnatal care; [31, 35] however, rates of maternal and neonatal mortality have not proportionally reduced. This disconnect between increased coverage a proportionality and slower reduction in mortality rates indicates that maternal and newborn services offered to women in Pakistan are not optimum. It is notable that, while the government set-up of health service has been considerable which endeavours to deliver healthcare at–primary, secondary, and tertiary levels, only 22% of total births occur in public health facilities [31] There are numerous reasons for this, including but not limited to, lack of technical competency of service providers a, critical shortage of health workforce, lack of supplies and equipment, lack of accountability and governance, and a condescending attitude of staff towards patients [36–38].

## Methods

### Study design and settings

A cross-sectional survey was conducted during August–November 2016 across four contiguous districts (Hyderabad, Jamshoro, Tando Muhammad Khan, Tando Allayar) of Sindh province in Pakistan. These districts are approximately 160 kilometres away from Karachi, which is the most populous metropolitan city of Pakistan

Our study focused on secondary healthcare which is mainly concerned with the provision of technical, therapeutic, and diagnostic services. Specialist consultation and hospital admissions fall into this category. It includes Tehsil Head Quarters (THQs), and District Health Quarters (DHQs). The THQs serve a population of 0.5 to 1 million people; whereas DHQs are located at the district level and services 1–3 million population. Both type of health facilities are supposed to offer basic and comprehensive Emergency, Obstetrtics and newborn care.

### Sample size determination

The sample size for the more extensive study was calculated based on an estimation formula using PASS version 11.0. Given that the reported prevalence of mistreatment during childbirth is varied significantly across studies owing to the differences in the operational definition of mistreatment, tools and methodology, a general 50% prevalence of mistreatment was anticipated which gives an optimal sample for estimation. Additionally, using a 5% margin of error and a 95% confidence interval, a sample of 385 was estimated. The sample was adjusted for possible correlation within hospitals (intra-cluster correlation) by using a design effect of 2. The number was further increased by adding 10% non-response, missing values, and lost-to-follow-up in home-based interview, which yielded a total sample of 790 women. We interviewed 783 women during the survey.

### Sampling strategy

At first, four districts were purposively selected in the light of practical constraints of time, budget, and logistics. Within these districts data were collected at six secondary-level public health facilities. Of those, two were district headquarters hospitals (DHQ) and four were tehsil headquarters hospitals (THQ). By using a consecutive sampling technique, eligible women who had given birth at the selected hospitals during the study period were invited to participate in the study. Data were simultaneously collected at all sites until desired sample was achieved; thus, the sample was self-weighted and proportional to the birthing volume at each hospital. Women residing in the same districts were invited to participate in the survey.

### Study questionnaire

A structured questionnaire was developed which comprised following sections: socio-economic and demographics characteristics, household decision making, social support, domestic violence, reproductive health (utilisation of antenatal and postnatal essential educational components). Each section in the structured questionnaire was adapted from a standardised version that has been previously used in Pakistan [26, 31, 39]. Section on mistreatment was developed in accordance with the standard WHO-framework published earlier by Bohren and colleagues [2] based on a mixed-method systematic review. The items were classified into: physical abuse, verbal abuse, stigma and discrimination, failure of meet standards (non-confidential, non-consensual care, neglect and abandonment), lack of supportive care, loss of autonomy, and healthy system conditions and contraints (see appendix 1 more details on items). The questionnaire has been pre-tested among 40 postnatal women using the cognitive pretesting method [40] before study data collection.

### Data collection

Trained enumerators collected the data through face-to-face interviews in privacy on paper-based forms. Sections related to socio-demographics and reproductive history were administered in hospital-based interviews when the woman had fully recovered after the delivery. Information on women’s experiences of mistreatment was collected in a home-based interview within 6-weeks of postpartum. On average, it took 10 and 40 minutes to conduct hospital- and home-based interviews respectively. Women were informed about 6-week home-based follow-up interview at the time of consent, those who gave voluntary informed consent were recruited in the study. All study participants gave written (thumbprint, in case of illiteracy) voluntary informed consent to participate in the study. Data were double-entered in Epidata version 3.1.

### Measures

#### Outcome variable

Experiences of mistreatment during childbirth was the outcome variable for this study. Construction of overall mistreatment measure and types of mistreatment were guided by WHO’s framework on mistreatment [2] as described in above under study questionnaire. The overall index of mistreatment was constructed based on 43 indicators that are identified as the strongest correlates of mistreatment (S1 Table) [41]. We constructed a composite index by adding the scores for each item after reverse coding of positively worded items. All indicators were dichotomous (0 or 1), where ‘1’ indicates ‘experienced mistreatment’, and ‘0’ ‘not experienced’. The total raw score that ranged from 0–43, which was linearly transformed on a scale of 0–100, where a higher score indicates a higher level of mistreatment. The transformed score was calculated in the form of a percentage which is the sum of all items scores out of the maximum possible score of 43. The same procedure was repeated for ‘individual composite indices’ for each type of mistreatment.

#### Independent variables

The independent variables were broadly classified as: (I) Socio-demographics, (II) Health Services, and (III) Womens’ empowerment. Below we described the construction of variables used for each category.

(I) Socio-demographics included age, primigravida, religion, women’s education, ethnicity, and wealth status. We used the wealth quintile as a primary measure of socio-economic position (SEP) which is widely used internationally, especially in demographic health surveys [42]. It is considered as a more robust SEP measure for lower-middle countries as opposed to income or consumption-based indices [27, 42]. The index was computed by using principal component analysis, based on information on household assets (electricity, radio, refrigerator, television, chairs, sofa, camera, sewing machine, car/truck/tractor, phone, air-conditioner, washing machine, bed, wall-clock, cupboard, motorcycle, engine-boat, and animal cart) and amenities (source of drinking water, the material used in household construction, fuel used for cooking, and toilet facility) [42]. The index was calculated through principal component using tetrachoric and polychoric correlation matrix given the binary and ordinal type of variables, respectively [43].

(II) Health Services category comprised of type of person assisted with childbirth, indices of health education on essential birth preparedness and postnatal/postpartum care for the index pregnancy, and mode of delivery. The index for women’s education on birth preparedness was based on six essential elements of care as recommended by WHO [44]: (a) at least four antenatal visits, (b) skilled birth delivery, (c) prior identification of skilled birth attendant, (d) transport arrangement, (e) recognising danger signs, (f) identification of resources for managing emergency (funds, communication), (g) identification of blood donor, and (h) keeping the baby warm. Women coded as ‘1’ if they were educated on any one element of birth preparedness and ‘0’ if they had received none. The same method was used for postnatal/partum index, based on whether women were educated on: (a) mother’s nutrition, (b) mother’s hygiene, (c) baby’s hygiene, (d) feeding of baby, (e) birth spacing (family planning), (f) immunisation, (g) recognising danger signs for mother, and (h) danger signs for the child. The scores for both the indices range from 0–8, where a higher score indicates receiving more education.

(III) Women’s empowerment comprised of intimate partner violence (marital control exercised by husband, history of emotional and physical abuse by family), employment status, women’s involvement in household decision making jointly with husband, and social support [45]. The measures of intimate partner violence (IPV) were created based on standard questions that are used in the Pakistan Demographic Health Survey [31] that broadly classified IPV as acts of marital control exercise by husband, physical abuse, and emotional abuse [31]. The score of marital control exercise by husband ranged from 0–5 that was based on the following: (i) jealous or angry if the woman talks to other men, (ii) accuse of being unfaithful, (iii) doesn’t permit to meet female friends, (iv) limit contact with family, and (v) insist knowing whereabouts. Similarly, the measure of emotional abuse comprised of three questions whether any family ever did any of the following to the women: (i) say or do anything to humiliate, (ii) ever threaten to hurt or harm women or someone close to women, and (iii) insult or make a woman feel bad about herself. Lastly, physical abuse comprises the following questions: (i) whether the husband ever hit, slapped, kicked, or done anything else to hurt physically and (ii) whether in-laws ever hit, slapped, kicked, or done anything else to hurt physically.

Moreover, the degree of women’s involvement in household decision making was measured through a standard inventory [39]. Women were asked who makes the final decision for the following: (1) number of children to have, (2) where to take her children in the event of illness, (3) where to go for medical care in the event of her illness, (4) children’s education, (5) small household expenditure (e.g. toothpaste, soap, crockery etc.), (6) significant household expenditure (e.g. TV, refrigerator, furniture etc.), (7) expenditure on her clothes, cosmetics, jewellery etc., (8) expenditure on children’s clothes, (9) expenditure on medicines, (10) buying or selling of property, (11) her employment outside the home, (12) visiting relatives, (13) whether she leaves the house alone for medical help, and (14) where to spend earned money. Decisions that are jointly made couple were coded as ‘1’ and ‘0’ otherwise. The score ranged from 0–15, with a higher score indicating a higher degree of involvement in household decision making. Information on social support was collected using Duke Social Support Scale and support scores were calculated following the standard scoring guidelines [45]. The scores range from 0–100, where a higher score indicates more significant social support for women. It is important to note that we didn’t create an overall measure of women empowerment but the effect of each measure was assessed separately in the analysis.

### Ethics

The institutional ethics committee of the International Center for Chemical and Biological Sciences, University of Karachi, Pakistan (Protocol No. ICCBS/IEC-017-S-2016/Protocol/1.0) approved the study protocol. Study participants were informed in detail about this research that included: research objectives, any real or perceived risks or benefits, the expected duration of an interview, the freedom to refuse to answer any question or to withdraw at any stage of the interview, and importantly assurances of confidentiality. All participants gave verbal consent before the interview.

### Statistical analysis

**Fig 1:**
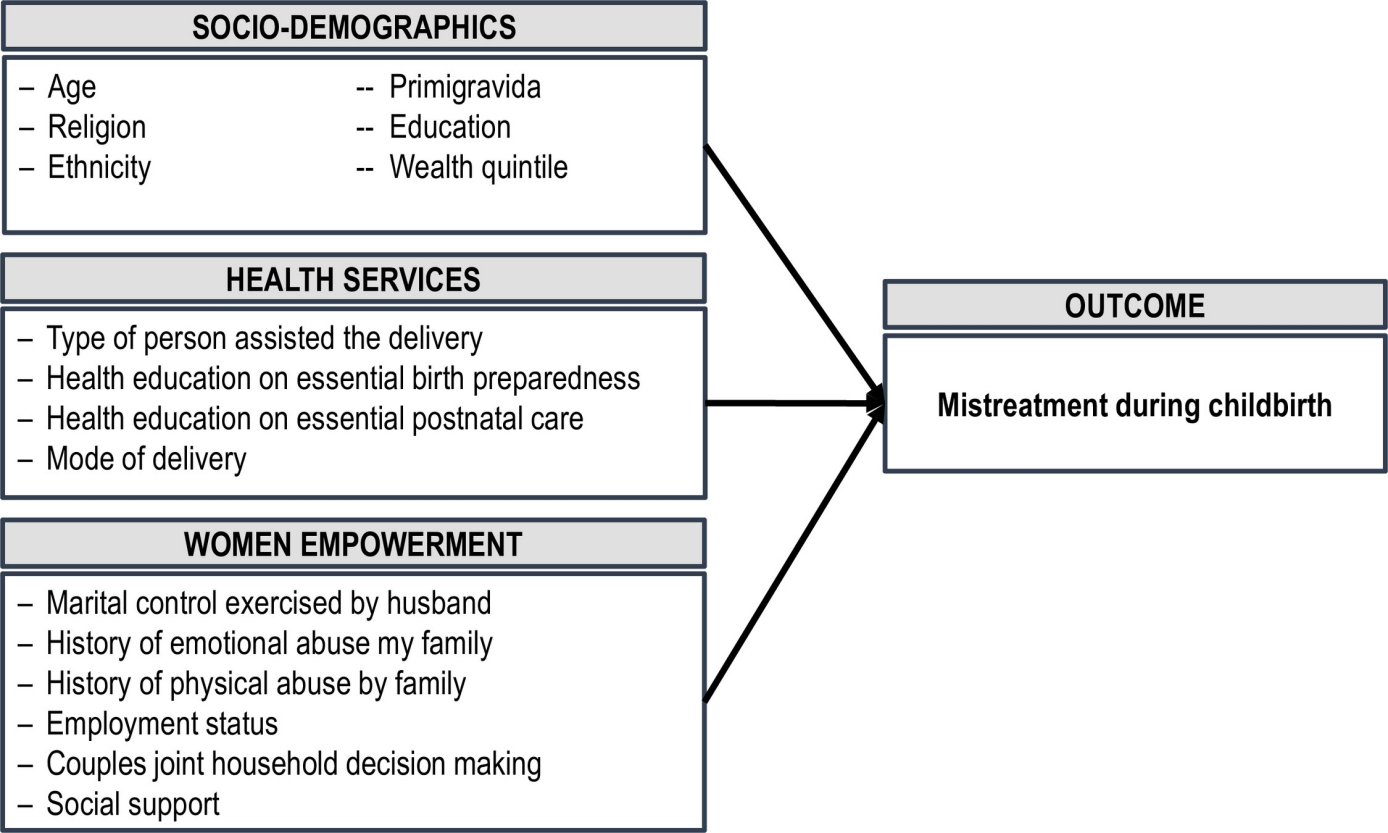
Analytical framework.

The analytical framework is presented in Fig 1 guided the analysis. All data analyses were performed using Stata version 15.1 (StataCorp. 2017. Stata Statistical Software: Release 15. College Station, TX: StataCorp LP). We used means and proportions to describe the characteristics of the study population. Kuder Richardson’s formula was used to assess the internal consistency of the overall 43 items mistreatment and for eight types of mistreatment. We also applied ordinary least square regression models to estimate the adjusted effect of each risk factor with reports of mistreatment. A p-value of 0.05 was considered statistically significant.

## Results

The characteristics of study participants are presented in Table 1. The mean age of women was 25.7 years and nearly one-third (35.8%) were primigravida. More than ninety percent were Muslim; and Muhajir (47.2%) and Sindhi (40.9%) were the most predominant ethnic groups. Approximately 37% of the women were illiterate and 54.3% had completed some level of primary education. Two in five women received no education on essential aspects of birth preparedness during the antenatal period whereas only 6% of the women reported having received no postnatal education. The majority (60.7%) of the women gave birth by caesarean. The physician-assisted were every four in five births.

**Table 1:** Characteristics of the study participants.

Socio-demographic	N / Mean	Percentage / SD	Health Services	N / Mean	Percentage / SD
Women’s age			Birth preparedness education		
Mean (±SD)	25.7	(± 5)	None	312	39.8
Primigravida			Mean (±SD)	2.2	2.4
Yes	281	35.8	Postnatal/partum care education		
No	503	64.2	None	46	5.9
Religion			Mean out of 8 (±SD)	3.9	2
Islam	729	93.1	Mode of birth (qh10)		
Hinduism	51	6.5	Normal	308	39.3
Christianity	3	0.4	Caesarean section	475	60.7
Women’s education			Person assisted the birth		
Illiterate	291	37.2	General physician	613	78.3
Some primary	426	54.4	Midlevel provider	170	21.7
Completed primary or higher	66	8.4	Hospital		
Ethnicity			District Head Quarter	236	30.1
Muhajir	370	47.3	Taluka Head Quarter	547	69.9
Punjabi	43	5.5			
Sindhi	321	41.0			
Balochi	23	2.9			
Pathan	26	3.3			

**Fig 2:**
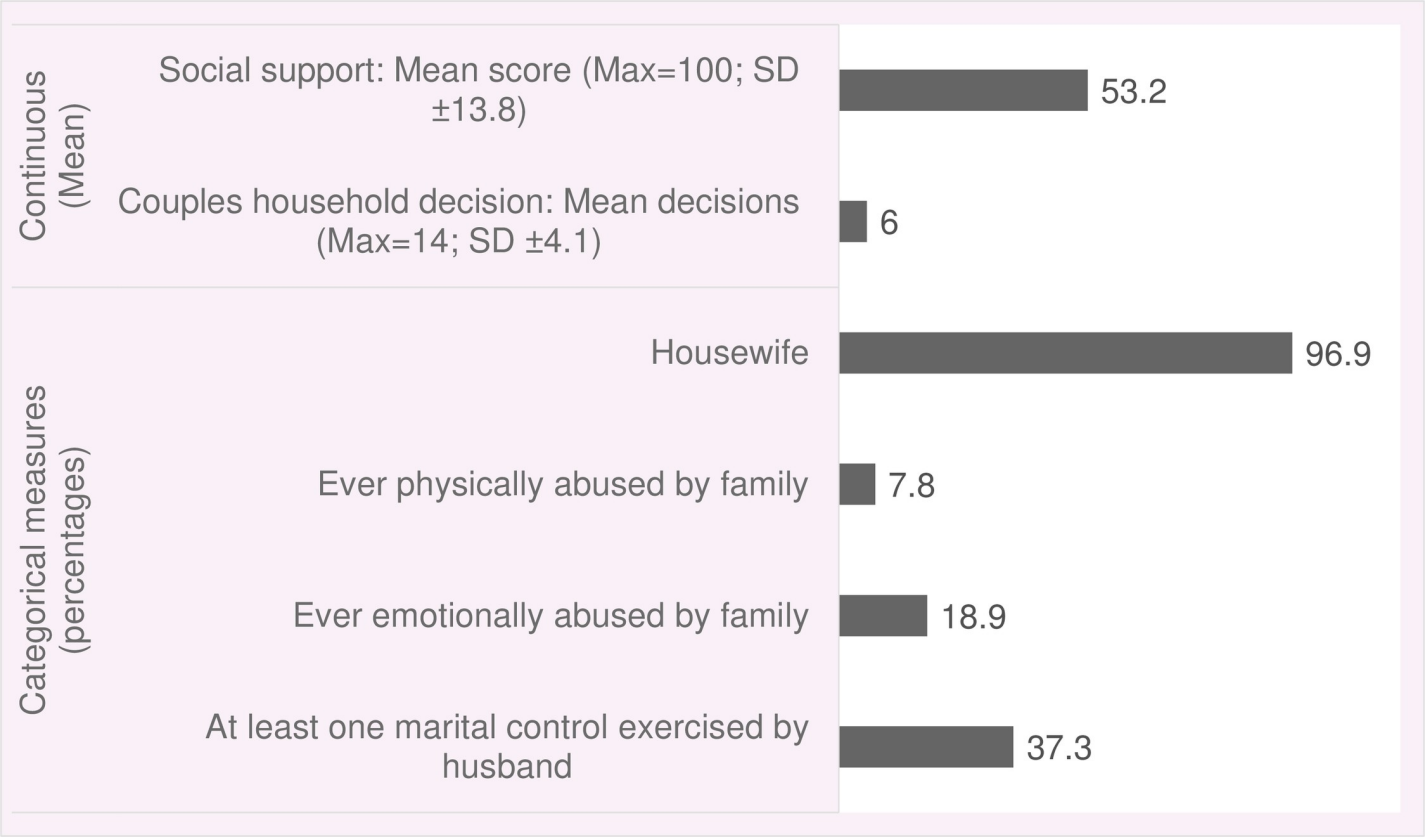
Measures of women’s empowerment.

Measures of women’s empowerment are presented in Fig 2. Ninety-seven percent of the women were housewives; on an average women reported to take 6 decisions (out of 13 items asked) jointly with husbands about household affairs. The proportion of women reported to be ever emotionally and physically abused by the family was 18.9% and 7.8%, respectively. Just over one-third (37.3%) of the women reported to experience at least one act of marital control exercised by their husbands.

**Fig 3:**
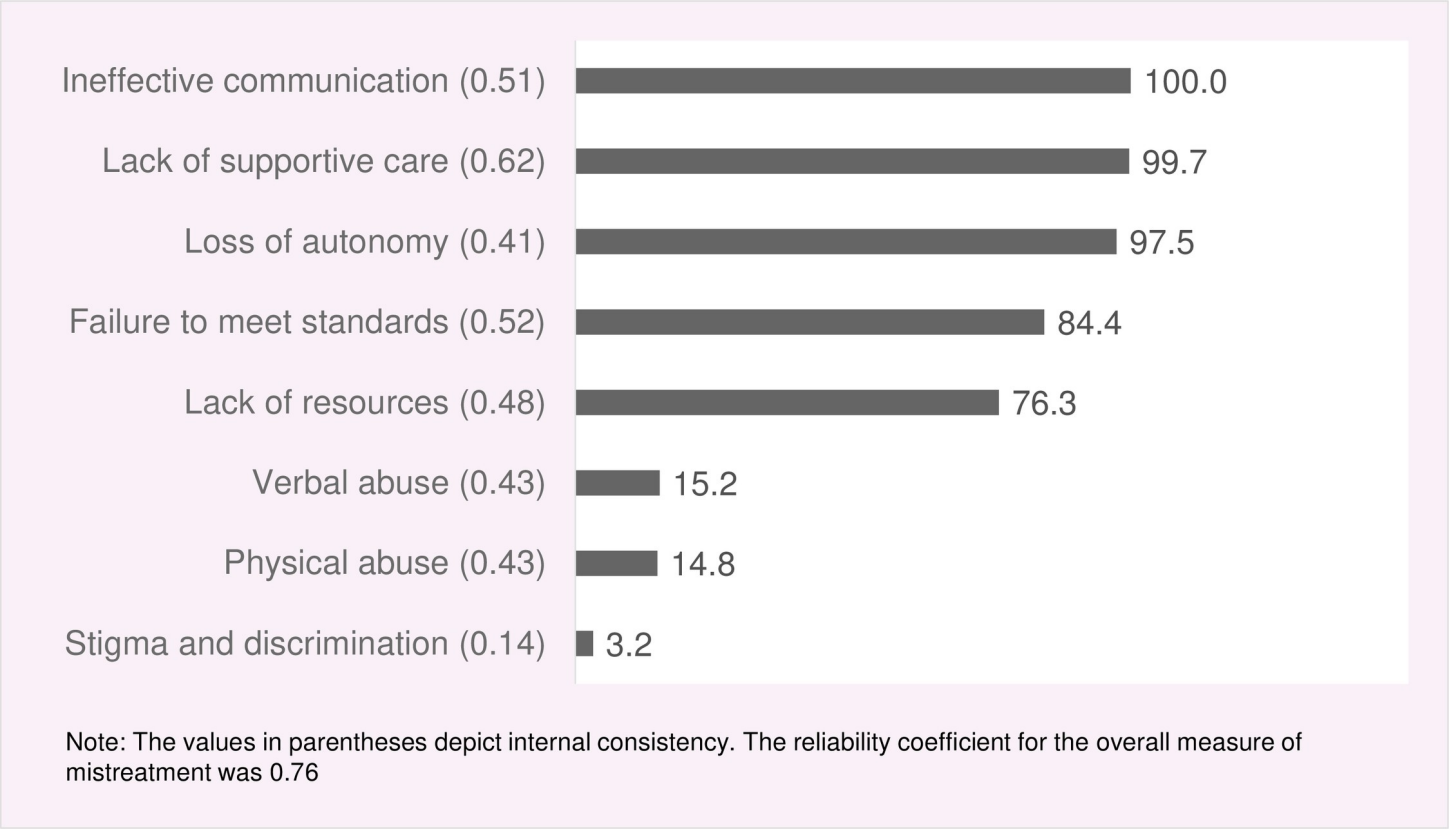
Prevalence of mistreatment during childbirth in facility-based settings, according to type of mistreatment.

The most common manifestations of mistreatment during childbirth in health facility were ineffective communication (100%), lack of supportive care (99.7%), and loss of autonomy (97.5%) followed by failure of meeting professional clinical standards (84.4%) and lack of resources (76.3%). About 15% of the women each reported experiences at least one manifestation of verbal and physical abuse, and only 3.2% reported experiencing discriminatory care (Fig 3).

**Fig 4:**
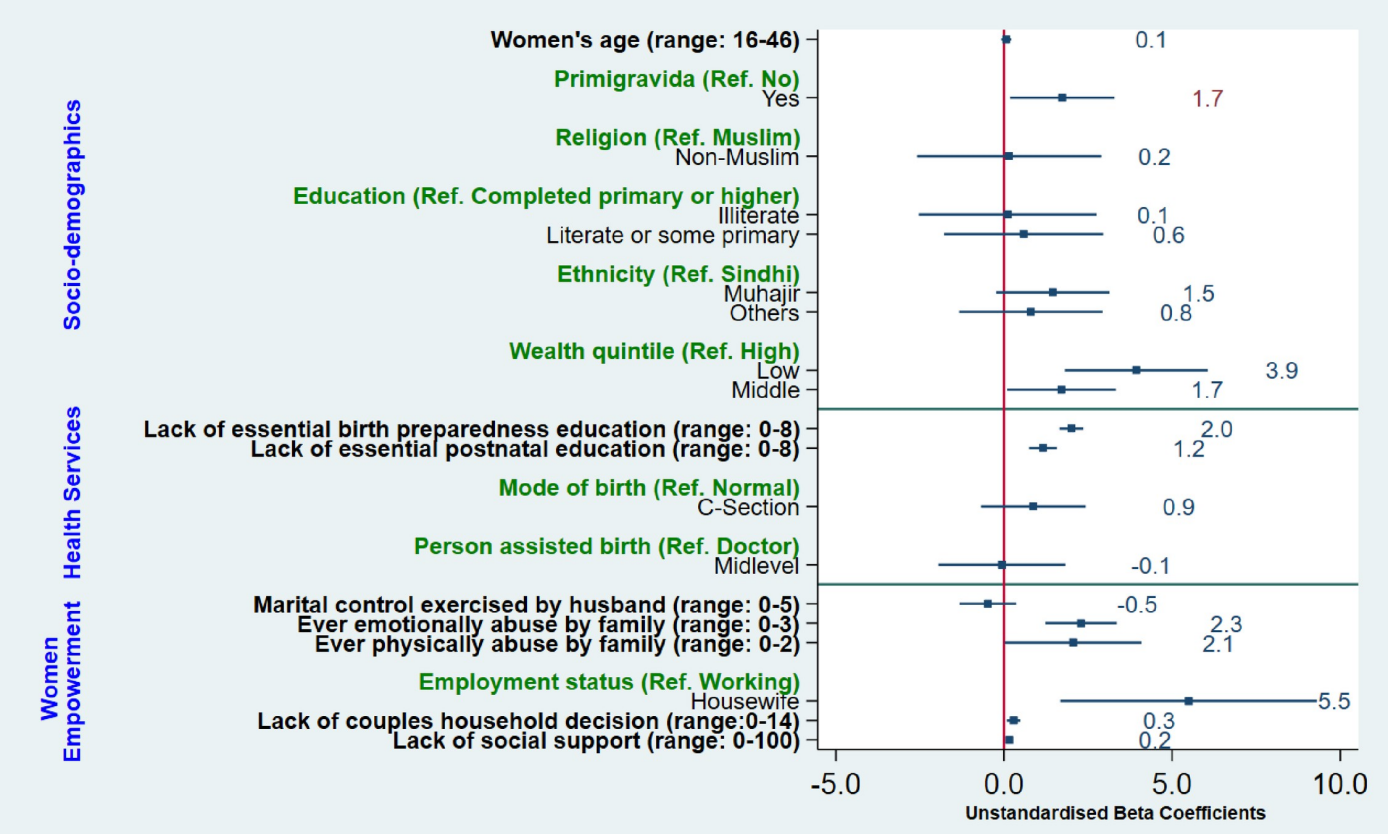
Factors underlying inequalities in women’s experiences of mistreatment during childbirth in facility-based settings.

Factors underlying inequalities in women’s experiences of mistreatment during childbirth are presented in Fig 4. Compared with affluent women, those belonged to middle (β = 1.7, 95% CI 0.09, 3.33; p-value = 0.04) and low (β = 3.9, 95% CI 1.81, 6.07; p-value<0.001) wealth quintile reported higher levels of mistreatment. Primigravida women were more (β = 1.7, 95% CI 0.17, 3.29; p-value = 0.03) vulnerable to be mistreated as opposed to multigravida. The lesser the women is educated about essential birth preparedness (β = 2.0, 95% CI 1.65, 2.36; p-value <0.001) and postnatal care (β = 1.16, 95% CI 0.75, 1.57; p-value <0.001), the higher the risk of mistreatment during childbirth.

Four out of five measures of women empowerment showed significantly association with mistreatment during childbirth. The more women are emotionally abused by family, the higher (β = 2.29, 95% CI 1.22, 3.35; p-value <0.001) mistreatment they encounter during childbirth. Similarly, experiences of mistreatment proportionally increase with experiences of physical abuse (β = 2.05, 95% CI 0.18, 4.09; p-value = 0.048). The lesser women are involved in household decision making with husband, higher the level of mistreatment during childbirth (β = 0.28, 95% CI 0.08, 0.48; p-value = 0.006). Women who were housewives/unemployed were more (β = 5.50, 95% CI 1.67, 9.32; p-value = 0.005) likely to be mistreated as compared with working women. Women who lack social support are more (β = 0.2, 95% CI 0.11, 0.22; p-value <0.001) likely to be mistreated.

## Discussion

Our study found a high prevalence of and stark inequalities in women’s experiences of mistreatment during childbirth in public health facilities. The high prevalence was consistent with previous studies conducted in Pakistan [26, 29], and other South Asian [46] and African [47] countries. It was higher than the reported prevalence of African countries such as Ethiopia [48], Kenya [49], and Tanzania [50].

In terms of different types of mistreatment, ineffective communication (100%), lack of supportive care (99.7%), and loss of autonomy (97.5%) were most prevalent, followed by failure to meet professional standards (84%). These findings indicate that the notion of women-centred care–requiring healthcare professionals to keep women involved in the process by informing them about progress and clarifying their doubts, ensuring confidentiality, providing necessary physical and emotional support, and giving them the autonomy to make informed choices about childbirth–is clearly being flouted [51–53]. Possible explanations for this are: service- provider’s lack of understanding of women’s rights [54], service providers’ failure to recognise that the manifestations of their behaviours amount to mistreatment of women [55], or the normalisation of those behaviours [56, 57].

Reports of verbal and physical abuse hovered at around 15% each which is similar to previous studies conducted in Pakistan [26, 29] but far lower than the reported figure of 42% from the most recent multi-country study led by WHO [3]. Studies conducted in African countries reported varied percentages ranging from 0.2% to 9% for physical abuse and 1.9% to 18.1% for verbal abuse [58–60]. Physical or verbal abuse are most likely around the time of birth [3]: this is- when care providers are present, and working under stress as they manage birthing and potential complications. Service providers rationalise such behaviours as ‘ punishing ‘ uncooperative women for the sake of preventing any adverse outcome for mothers and baby [57, 61]. However, not only does such abuse violate clinical protocols but it can also inflict physical or psychological suffering on women [11, 12, 62].

### Inequalities based on socio-demographics characteristics

Contrary to the documented findings of other studies [2, 13], age, religion, ethnicity, and education showed no effect on how women are treated during childbirth in public health facilities. There are various possible reasons of this contrastOur analysis included a variable on the history of pregnancy which showed that primigravida women are more mistreated than multigravida mothers. This indicates that a lack of prior exposure to childbirth perhaps is a stronger determinant of mistreatment than merely being young. Anxiety and fear of birth are higher among primigravida—- this likely results in a greater need for care, which may be resented by care providers hence resulting in mistreatment [63]. Conversely, the prior experience of multigravida may have normalised mistreatment, resulting in their under-reporting of it. Lack of variability in religion (~93% being Muslim) and ethnicity (>90% being Sindhi and Mujhar) combined with a limited sample size constrained our options for exploring how women with different religions and ethnic backgrounds are treated. Education is often used as a proxy for empowerment, We observed no relationship between education and levels of mistreatment: this, we believe, is possibly due to the inclusion of separate measures of women’s empowerment in our analysis that may have diluted the education-mistreatment relationship. In keeping with other studies [50, 64], we found that being poor significantly increases the risk of mistreatment, probably due to class-based discrimination and service providers’ beliefs that poor women lack the power or privileges to retaliate [65].

### Inequalities based on women empowerment characteristics

Notably, five out of six measures of women’s empowerment showed an independent association with mistreatment. This clearly signifies that an increased level of empowerment substantially reduces the risk of mistreatment as elicited in a study from India [25]. Women who had ever experienced emotional or physical abuse by their family reported higher levels of mistreatment. Research suggests that social norms around violence against women are associated with how women are treated in health facilities, including during childbirth [66, 67]. Acceptance of violence against women at individual and community levels may influence how women are treated in a facility by care providers, their acceptance of that treatment, and how women are supported by the health system in general [49, 66]. Mistreatment of these women can cause their physical and mental condition to further deteriorate, in terms of feeling pain, re-traumatisation, anxiety, and feeling dehumanised [9–12]. We, therefore, suggest that psychological support is needed for emotionally disturbed women during maternity care. Housewives are more vulnerable than working women to the risk of mistreatment In contrast to the previous study [26], women’s lack of involvement in household decision making increases the risk of mistreatment. Consistent with another study from India [25], social support had a protective effect on mistreatment. This relationship resonates with the WHO’s concept of respectful maternity care that reinforces the provision of social support to women for improved birthing experiences [8]. A woman’s experience of social support may also influence her personal sense of empowerment; either that, or her companion may have advocated for her, thus helping her receive better care [68]. Overall, women’s empowerment is positively associated with awareness of rights and an increased level of self-confidence that could help minimise the power differential with service providers, consequently reducing the likelihood of being mistreated.

### Health services factors and mistreatment

Women who were less-educated in essential aspects of birth preparedness reported higher levels of mistreatment. This finding is consistent with the previous study conducted in Pakistan [26] and with interventional research in Tanzania, which found a protective effect of birth-preparedness education on mistreatment [69]. Our study also noted that mistreatment and education on essential postnatal care go hand-in-hand–i.e., the more women are mistreated the less educated they are in postnatal care. This finding indicates that certain sections of the population are not more mistreated in birthing facilities, but rather their mistreatment is part of the holistic sub-standard care that they recieve.

### Strengths and limitations

The main strengths of our study are the prospective recruitment of women in public health facilities and gathering sensitive information about their maternity care experiences in home-based interviews within six -weeks postpartum. This reduces the element of recall and courtesy bias. To minimize the risk of confounding bias, our study simultaneously accounted for a range of potential health system and socio-economic and demographic risk factors that had not previously been studied together previously. Furthermore, most of the complex and multidimensional measures used in our study were constructed based on several questions rationalised from reliable empirical literature, which addeds to the study’s rigour. The composite index of mistreatment was also based on items that had been identified through literature and advanced statistical analysis [41]. In terms of limitations, the study relied solely on women’s reports of mistreatment;- and other studies have shown a discrepancy between the results of direct observation and women’s reported experiences [50]. There is a likelihood of women underreporting mistreatment as some aggravating behaviours may have become normalised. The information on perceived social support was collected during post-partum interviews. In our opinion, the level of support may vary across during the antenatal, intrapartum, and postpartum periods. However, the measure gives proximate information on how social support may prevent mistreatment during childbirth. Lastly, using the etic approach the study focused only on the experiential aspect of mistreatment and did not account for the subjective views of participants. Evidence shows that in certain circumstances birthing women deem disrespectful behaviours acceptable, or even appropriate in that they ensure positive birth outcomes [57, 70].

## Conclusion

In summary, we found high prevalence of mistreatment during childbirth in public health and glaring inequalities in terms of the way women are treated during childbirth in public health facilities. In a nutshell, measures of socio-demographic, health services, and women’s empowerment showed a significant independent association with mistreatment during childbirth. Protective effect of almost all multidimensional measures of women’s empowerment on mistreatment signifies the need for–holistically promoting women’s empowerment such as preventing intimate partner violence, involvement in household decision making, increased social support, and financial autonomy by allowing them to earn for their families. At the health system level, there is a need for urgent solutions for more inclusive care to ensure that all women are treated with compassion and dignity, complemented by psychosocial support for those who are emotionally disturbed and lack social support. More research is urgently needed to have a deeper understanding of health system issues that drive disrespect and abuse and identifying the opportunities to inculcate behavioural and systemic changes for women-centred care.

## Supporting information

S1 Table Indicators for mistreatment during childbirth in health facility, according to type of mistreatment. (DOCX)
