# Supplementary material for: Are underprivileged and less empowered women deprived of respectful maternity care: Inequities in childbirth experiences in public health facilities in Pakistan
Source: PLoS One. 2021 Apr 15;16(4):e0249874. doi: 10.1371/journal.pone.0249874 (PMC8049770; doi:10.1371/journal.pone.0249874)
Supplement: S1 Table — (DOCX) [file pone.0249874.s001.docx]

| \| **S1 Table. Indicators for mistreatment during childbirth in health facility, according to type of mistreatment** \| \| --- \|  \| **S.no** \| **Measures** \| **Items** \| **Score range** \| \| --- \| --- \| --- \| --- \| \| 1 \| Physical abuse \| (a) pinch irritably, (b) push badly to change position, (c) slapping, (d) excessive force to pull baby; \| 0 - 4 \| \| 2 \| Verbal abuse \| (a) threaten for negative consequences, (b) harsh tone, yelling or shouting, (c) abusive language, (d) pass insulting, accusatory or degrading comments \| 0 - 4 \| \| 3 \| Stigma and discrimination \| (a) perceived poor service due to ethnicity, (b) perceived poor service due to medical illness \| 0 - 2 \| \| 4 \| Failure to meet clinical standards (non-confidential, consensual care, and neglect and abandonment) \| (a) privacy during examination and procedure, (b) cover woman while taking to and from the birthing area, (c) permission for physical examination, (d) perform procedure without consent, (e) abandon women during labour, childbirth or afterward, (f) ignore while asking pain relief/medication without explanation \| 0 - 6 \| \| 5 \| Ineffective communication \| (a) introduce to the woman, (b) call woman by name, (c) explain examination, (d) provided too little information, (e) explain about operative procedure to be used for delivery, (f) explain type of procedure with reason, (g) explain what to expect from labour or childbirth, (h) regularly share progress of labour, (i) share progress of labour with companion, (j) encourage to ask questions, (k) explain when to contact in case of need \| 0 - 11 \| \| 6 \| Lack of supportive care \| (a) give choice of birth companion, (b) encourage woman or companion to stay together, (c) inform about toileting facilities, (d) praise and reassure woman for abiding instructions, (e) listen attentively, (f) assist woman to ambulate during labour, (g) comfort or hasten labour by breathing \| 0 - 7 \| \| 7 \| Loss of autonomy \| (a) interrupt woman while speaking, (b) denial woman’s preferred birth position, (c) offer choice for birthing procedure, (d) consult for discharge; \| 0 - 4 \| \| 8 \| Health system condition and constraints \| (a) sharing bed, (b) poor bed condition, (c) no or unclean bed linen, (d) provision of wheelchair or stretcher when needed \| 0 - 4 \| \| 9 \| Overall mistreatment \| All items as described above \| 0 - 43 \| \|  \|  \|  \|  \| |
| --- | --- | --- | --- | --- | --- | --- | --- | --- | --- | --- | --- | --- | --- | --- | --- | --- | --- | --- | --- | --- | --- | --- | --- | --- | --- | --- | --- | --- | --- | --- | --- | --- | --- | --- | --- | --- | --- | --- | --- | --- | --- | --- | --- | --- | --- |
